# Affirming the Value of the Resident Assessment Instrument: Minimum Data Set Version 2.0 for Nursing Home Decision-Making and Quality Improvement

**DOI:** 10.3390/healthcare3030659

**Published:** 2015-07-30

**Authors:** Lindsay S. Drummond, Susan E. Slaughter, C. Allyson Jones, Adrian S. Wagg, Melissa Batchelor-Murphy

**Affiliations:** 1 Department of Medicine, Faculty of Medicine and Dentistry, University of Alberta, Edmonton, AB T6G 1C9, Canada; E-Mails: lindsay.drummond@ualberta.ca (L.S.D.); adrian.wagg@ualberta.ca (A.S.W.); 2 Faculty of Nursing, Edmonton Clinic Health Academy, University of Alberta, Edmonton, AB T6G 1C9, Canada; 3 Department of Physical Therapy, Faculty of Rehabilitation Medicine, University of Alberta, Edmonton, AB T6G 1C9, Canada; E-Mail: cajones@ualberta.ca

**Keywords:** RAI-MDS 2.0, FIM, activities of daily living, dementia, nursing home

## Abstract

*Background:* We examined the agreement over time of the physical functioning domains of the Resident Assessment Instrument: Minimum Data Set Version 2.0 (RAI-MDS) and the Functional Independence Measure (FIM) in nursing home residents with dementia. *Methods:* We completed a secondary analysis of data from a longitudinal quasi-experimental study of residents who could transfer independently or with the assistance of one person. FIM assessments were completed at up to three time points by researchers using interviews. RAI-MDS assessments, completed by nursing home staff, were matched to the FIM assessment by nearest time. FIM and RAI-MDS assessments were correlated based on time between assessments using Pearson’s correlation. Items for activities of daily living (ADL) from the RAI-MDS were rescaled using two previously published crosswalks. Motor and ADL subscales were also used, containing eight and six items, respectively. *Results:* A total of 362 paired interviews and assessments were collected from 130 residents. The mean scores and standard deviations were as follows: FIM: 19.64 (7.60); William’s RAI-MDS crosswalk: 18.04 (5.25); and Velozo’s RAI-MDS crosswalk: 18.09 (6.50). Using both crosswalks, most items showed medium (r > 0.3) or large (r > 0.5) correlations, even at greater than 41 days between assessments. Subscales showed large correlations for all time intervals for both crosswalks. *Conclusions*: The RAI-MDS remains stable when data are collected greater than 41 days from the FIM assessment. These findings should add confidence in the RAI-MDS data and its clinical utility.

## 1. Introduction

Clinicians and administrators in nursing homes often rely on data collected on the functional status of residents to inform decisions to improve the quality of life of residents and to facilitate quality improvement initiatives. Because dependency in activities of daily living (ADL) is proportional to the amount of support required, it is important for administrators to have accurate data on the ADL of nursing home residents with dementia. The Resident Assessment Instrument: Minimum Data Set Version 2.0 (RAI-MDS 2.0) is a standardized instrument for data collection used internationally in nursing homes for care planning, reimbursement and quality monitoring [1] and contains over 300 items related to function, cognition and behaviour. While an adaptation of the RAI-MDS 2.0, the MDS 3.0 has been released, it is currently only used in the United States and version 2.0 continues to be used in other countries [2]. Often, data collected from residents in a nursing home can be aggregated to provide quality indicators about the care provided at the individual and facility levels and some researchers have used the RAI-MDS 2.0 to derive such indicators [2,3]. Quality indicators can help alert clinicians and administrators of potential areas of concern and, along with careful observation and investigation, help direct quality improvement in nursing homes.

Because the RAI-MDS 2.0 is gathered quarterly, administrators may question how long from the date of assessment the data remain valid for use, especially considering the progressive functional decline often encountered in residents with dementia [4]. The Functional Independence Measure (FIM) is a well validated and widely used measure of the burden of care containing 18 items related to physical function and cognition [5]. Crosswalks between the FIM and RAI-MDS 2.0 instruments have been developed using qualitative and quantitative analysis [6,7]. Crosswalks allow for better comparisons of residents in different types of facilities and are valuable to help understand the results of one instrument by users of another [5].

Previous studies have examined the reliability and validity of the RAI-MDS 2.0 [5], but not its agreement with FIM over time and when performed remotely from the RAI-MDS 2.0 assessment. Using secondary data gathered from the CIHR-funded MOVE study, described below [8], this study examined the correlation of individual RAI-MDS 2.0 and FIM data elements as well as Motor and ADL subscales created by Williams [7] at three time points, and compared these correlations by time between assessments.

## 2. Experimental Section

### 2.1. Study Design

The primary study was a quasi-experimental design which examined the effect of a mobility intervention in nursing home residents with dementia. The complete protocol summary is published elsewhere [9]. Participating residents had a diagnosis of dementia as obtained from their health records. Eligible participants were able to transfer independently, or with the assistance from one person. Informed written consent was obtained from residents or their authorized representatives. Ethics approval was granted by the Health Research Ethics Board at the University of Alberta.

FIM data, which was used to assess the burden of care for patients of multiple diagnoses [5], were collected by trained research assistants at a maximum of three time points using interviews with health care aides who worked directly with 130 residents in seven nursing homes. RAI-MDS 2.0 assessments were completed quarterly for all residents. RAI-MDS 2.0 data collection varied slightly between facilities; administrators reported that nursing home staff, including registered nurses, occupational therapists, dieticians and recreation therapists completed RAI-MDS 2.0 assessments for residents with ultimate responsibility lying with the registered nurse. Health care aides completed a 7-day tracking tool that was used to inform the completion of some RAI-MDS 2.0 sections, particularly those related to physical functioning.

### 2.2. Crosswalks

Crosswalks and rescales were created between FIM and RAI-MDS 2.0 items using methods described by Velozo [6] and Williams [7] (referred to as V-RAI and W-RAI, respectively). Williams matched FIM and RAI-MDS 2.0 items using an expert panel, while Velozo matched items with face validity and confirmed the matches with Rasch analysis. Both rescaled the direction of the RAI-MDS 2.0 scales so that higher numbers reflected greater independence and changed items from a 5-point to a 7-point scale. Because the original MDS used by Williams had a scale from 1 to 5 and the updated version 2.0 is scaled from 0 to 4, we adjusted the scale to reflect this revision. Williams matched 8 items and Velozo matched 9 (Table 1). Four notable differences between the crosswalks were: (1) Velozo included “Walk in Room -Walk/Wheelchair”; (2) Williams combined the more dependent score in FIM dressing upper or lower body and Velozo used upper body only; (3) Velozo excluded data matches that were outside the 95% confidence interval of differences between FIM and RAI-MDS 2.0 data; and (4) Williams included two summary scales: ADL and Motor. We also calculated ADL and Motor subscales on Velozo’s crosswalk for comparison to Williams’.

**Table 1:** Alignment of Functional Independence Measure (FIM) and MDS Items Used by Williams and Velozo.

FIM Item	MDS Item
Eating	Eating—Self Performance
Transfers: Bed, Chair, Wheelchair	Transferring—Self Performance
Toileting	Toilet Use—Self Performance
Bathing	Bathing—Self Performance
More dependent of: Dressing Upper Body, Dressing Lower Body	Dressing—Self Performance
Grooming	Personal Hygiene—Self Performance
Bladder Management	Bladder Continence
Bowel Management	Bowel Continence
Locomotion: Walk/Wheelchair	Walk in Room

### 2.3. Correlations

Research staff linked RAI-MDS 2.0 assessments to the nearest FIM interview by time for comparison. Times between RAI-MDS 2.0 and FIM assessments were categorized into eight, 7 day intervals based on the observation period for the RAI-MDS 2.0 (Table 2). Pearson’s correlation coefficients (*r*) were used to assess the FIM data and rescaled RAI-MDS 2.0 data. Statistical significance was defined at the *p* < 0.05 level. Correlations were interpreted using Cohen’s standards: small (*r* > 0.10), medium (*r* > 0.30) or large (*r* > 0.50) [10].

**Table 2:** Pearson’s correlation coefficients for comparisons between FIM and V-RAI assessments, and FIM and W-RAI assessments by time intervals between the assessments.

FIM Items & Subscales	0–6 Days	7–13 Days	14–20 Days	21–27 Days	28–34 Days	35–41 Days	>41 Days	All Days
**FIM and V-RAI**								
	*n* = 57	*n* = 60	*n* = 60	*n* = 50	*n* = 46	*n* = 24	*n* = 46	*n* = 343
Motor Subscale (8 Items)	0.78 **^‡^**	0.60 **^‡^**	0.64 **^‡^**	0.64 **^‡^**	0.76 **^‡^**	0.81 **^‡^**	0.72 **^‡^**	0.70 **^‡^**
ADL Subscale (6 Items)	0.83 **^‡^**	0.75 **^‡^**	0.74 **^‡^**	0.73 **^‡^**	0.82 **^‡^**	0.84 **^‡^**	0.77 **^‡^**	0.78 **^‡^**
Eating	0.58 **^‡^**	0.31 *	0.52 **^‡^**	0.53 **^‡^**	0.66 **^‡^**	0.38	0.58 **^‡^**	0.53 **^‡^**
Transfers	0.47 **^‡^**	0.52 **^‡^**	0.56 **^‡^**	0.42 **^†^**	0.59 **^‡^**	0.46 *	0.65 **^‡^**	0.52 **^‡^**
Toileting	0.67 **^‡^**	0.44 **^‡^**	0.33 **^†^**	0.64 **^‡^**	0.71 **^‡^**	0.87 **^‡^**	0.61 **^‡^**	0.60 **^‡^**
Bathing	0.53 **^‡^**	−0.14	−0.07	0.21	0.36 *	0.33	0.32 *	0.22 **^‡^**
Dressing	0.58 **^‡^**	0.44 **^‡^**	0.50 **^‡^**	0.36 *	0.49 **^†^**	0.75 **^‡^**	0.47 **^†^**	0.50 **^‡^**
Grooming	0.42 **^†^**	0.40 **^†^**	0.43 **^†^**	0.50 **^‡^**	0.53 **^‡^**	0.62 **^†^**	0.34 *	0.45 **^‡^**
Bladder Mgmt.	0.58 **^‡^**	0.52 **^‡^**	0.63 **^‡^**	0.78 **^‡^**	0.55 **^‡^**	0.53 **^†^**	0.66 **^‡^**	0.61 **^‡^**
Bowel Mgmt.	0.47 **^‡^**	0.56 **^‡^**	0.51 **^‡^**	0.48 **^‡^**	0.41 **^†^**	0.61 **^†^**	0.45 **^†^**	0.49 **^‡^**
Walk-Wheelchair	0.53 **^‡^**	0.56 **^‡^**	0.45 **^‡^**	0.63 **^‡^**	0.53 **^‡^**	0.58 **^†^**	0.23	0.50 **^‡^**
**FIM and W-RAI**								
	*n* = 59	*n* = 64	*n* = 63	*n* = 53	*n* = 48	*n* = 28	*n* = 47	*n* = 362
Motor Subscale (8 Items)	0.69 **^‡^**	0.52 **^‡^**	0.40 **^‡^**	0.58 **^‡^**	0.75 **^‡^**	0.64 **^‡^**	0.67 **^‡^**	0.61 **^‡^**
ADL Subscale (6 Items)	0.71 **^‡^**	0.62 **^‡^**	0.49 **^‡^**	0.62 **^‡^**	0.78 **^‡^**	0.68 **^‡^**	0.74 **^‡^**	0.67 **^‡^**
Eating	0.57 **^‡^**	0.36 **^†^**	0.49 **^‡^**	0.51 **^‡^**	0.65 **^‡^**	0.32	0.55 **^‡^**	0.50 **^‡^**
Transfers	0.50 **^‡^**	0.50 **^‡^**	0.46 **^‡^**	0.37 **^†^**	0.60 **^‡^**	0.41 *	0.61 **^‡^**	0.48 **^‡^**
Toileting	0.57 **^‡^**	0.34 **^†^**	0.24	0.56 **^‡^**	0.65 **^‡^**	0.71 **^‡^**	0.61 **^‡^**	0.54 **^‡^**
Bathing	0.42 **^†^**	−0.13	0.07	0.22	0.38 **^†^**	0.23	0.38 **^†^**	0.19 **^‡^**
Dressing	0.55 **^‡^**	0.36 **^†^**	0.34**^†^**	0.52 **^‡^**	0.57 **^‡^**	0.82 **^‡^**	0.48 **^†^**	0.50 **^‡^**
Grooming	0.34 **^†^**	0.32 **^†^**	0.27 *	0.41 **^†^**	0.48 **^†^**	0.43 *	0.23	0.36 **^‡^**
Bladder Mgmt.	0.44 **^†^**	0.45 **^‡^**	0.46 **^‡^**	0.56 **^‡^**	0.51 **^‡^**	0.36	0.62 **^‡^**	0.50 **^‡^**
Bowel Mgmt.	0.40 **^†^**	0.45 **^‡^**	0.48 **^‡^**	0.39 **^†^**	0.40 **^†^**	0.55 **^†^**	0.44 **^†^**	0.44 **^‡^**

Significance: * (*p* < 0.05); **^†^** (*p* < 0.01); **^‡^** (*p* < 0.001). Motor Subscale includes: Eating, Transfers, Toileting, Bathing, Dressing, Grooming, Bladder Management and Bowel Mgmt. ADL Subscale includes: Eating, Transfers, Toileting, Bathing, Dressing and Grooming.

## 3. Results

A total of 362 paired assessments were collected from 130 residents. Residents had a baseline mean age of 86.3 years (SD = 7.27) and 70.8% (*n* = 94) were female. No statistical differences (*p* < 0.05) were seen between time interval groups with respect to age and sex. Nineteen (15%) residents (mean age of 87.7 (SD = 8.42)) did not complete the study due to death or transfer to another institution. In addition, 19 (5.2%) of the 362 paired assessments were removed from the V-RAI comparison as per Velozo’s methods because they were outside the 95% confidence interval for difference between RAI-MDS 2.0 and FIM data.

The mean scores and standard deviations for the total FIM, W-RAI and V-RAI were 19.64 (7.60), 18.04 (5.25) and 18.09 (6.50), respectively. Correlations between FIM and V-RAI ranged from −0.068 to 0.856 and 92% were considered medium or large and statistically significant, with the exception of Eating at 35–41 days (r(24) = 0.38, *p* = 0.06) and RAI Walk in Room at >41 days (r(46) = 0.23, *p* = 0.13) (Table 2). Aggregated Motor (r(343) = 0.70, *p* < 0.001) and ADL (r(343) = 0.78, *p* < 0.001) subscales showed the highest levels of association for all time intervals. Correlations between FIM and W-RAI ranged from 0.07 to 0.82 and 88.1% and were medium or large and statistically significant, but generally smaller than correlations between FIM and V-RAI (Table 2). Small or negligible correlations included the following subscales: Eating at 35–41 days (r(28) = 0.32, *p* = 0.10), Toileting at 14–20 days (r(63) = 0.24, *p* = 0.06), Grooming at 14–20 days (r(63) = 0.27, *p* = 0.03), Grooming at >41 days (r(47) = 0.23, *p* = 0.12) and Bladder Management at 35–41 days (r(28) = 0.36, *p* = 0.06). For both crosswalks, correlations for bathing were inconsistent and did not reach statistical significance at any of the four intervals.

## 4. Discussion

Within this cohort of nursing home residents with dementia, correlations between the FIM and V-RAI, and FIM and W-RAI remained relatively consistent over time, even with greater than 41 days between assessments, reflecting good agreement of the resident level RAI-MDS 2.0 data. This finding suggests that administrators can confidently use RAI data to inform decision making or facility quality improvement. The use of motor and ADL subscales is recommended because correlations for these were larger than individual items.

Some inconsistencies were observed; correlations with V-RAI and FIM items were slightly larger and had a higher number of significant correlations, likely because Velozo excluded assessments which were outside the 95% confidence interval of the difference between FIM and RAI-MDS 2.0 data. Correlations not significant at 35–41 days may be accounted for by the smaller sample size at these time intervals. Correlation between RAI-MDS 2.0 and FIM bathing items was not significant at four time intervals, possibly because of the different definitions used for the bathing items. The RAI-MDS 2.0 includes tub transfers in bathing, while the FIM has a separate item for tub transfers. Because it is standard practice in nursing homes to assist with transfers into the tub or shower, nearly all residents score higher on the RAI-MDS 2.0 because of the assistance they receive.

Correlations on the Motor and ADL subscales for all time intervals were slightly lower than those reported by Velozo (r(236) = 0.78) and Williams (r(173) = 0.72) [6,7]. This may be due to the different methods used for the FIM and the RAI-MDS 2.0 data collection. RAI-MDS 2.0 data were collected by nursing home staff by variable methods in sites. In practice, the accuracy of RAI-MDS 2.0 data may be limited because of time constraints, staff turnover and paperwork burden [11], whereas FIM data were gathered by trained research assistants who were assessed for inter-rater reliability.

The method of collection of the RAI-MDS 2.0 data is both a strength and a limitation of this study. Although the inconsistent approach to data collection may compromise the internal validity of the study, the strength of correlation with the crosswalks found here and the agreement of the RAI-MDS 2.0 data over time are reassuring for nursing home administrators and clinicians who use RAI-MDS 2.0 data in practice.

## 5. Conclusions

The RAI-MDS 2.0, an internationally recognized instrument, remains relatively stable for use with nursing home residents with dementia even if data are used 41 days after collection. These findings affirm the value of the RAI-MDS 2.0 for nursing home decision-making and quality improvement.
